# Emotional intelligence among Tunisian medical residents

**DOI:** 10.1192/j.eurpsy.2025.1636

**Published:** 2025-08-26

**Authors:** H. Daoud, I. Sellami, A. Feki, M. L. Masmoudi, K. Jmal Hammami, M. Hajjaji

**Affiliations:** 1 Department of Occupational Medicine, Hedi Chaker Hospital; 2 LR/18/ES-28, Sfax university; 3 Department of rheumatology, Hedi Chaker Hospital, Sfax, Tunisia

## Abstract

**Introduction:**

Emotional intelligence (EI) has gained increased attention in medical education and research. EI is often defined as the ability to perceive, express, understand, and manage émotions making it a crucial skill to cultivate during medical school.

**Objectives:**

This study aimed to explore levels of global EI among Tunisian medical residents based on their demographic characteristics.

**Methods:**

This cross-sectional study was carried out with medical residents in training at hospitals across Tunisia. We gathered data anonymously through a Google Forms survey conducted from October 2023 to January 2024. Participants first completed a sociodemographic questionnaire, followed by the Trait Emotional Intelligence Questionnaire-Short Form (TEIQue-SF). This 30-item assessment measures overall trait emotional intelligence and evaluates four specific dimensions : Well-Being, Self-Control, Emotionality, and Sociability.

**Results:**

Our study included 127 participants, with men comprising one-fourth (25.2%). The mean age was 27.24±1.34 years with majority aged under 30 (93.7%). The majority of participants were single (81.8%). Most participants (71.7%) were pursuing a medical specialty. First-year medical residents represented 39.4%, while fifth-year residents made up only 1.2%. The total EI score was 4.6±0.68. The mean scores for the four EI traits factors were as follows : well-being 4.81±1.07, self-control 4.34±0.9, emotionality 4.86±0.73, and sociability 4.45±0.85.

Univariate analysis showed that higher levels of global EI, self-control, and sociability were associated with the male gender, with p= 0.027, p=0.00 and p=0.02 respectively. Also, final-year residents demonstrated significantly lower emotionality scores compared to first-year medical residents. Moreover, participants in medical specialties were significantly associated with higher well-being scores (p = 0.017).

**Conclusions:**

It is crucial to further investigate the factors contributing to these variations in EI and to develop tailored strategies that can effectively enhance EI among medical professionals and to support its development in the next generation of physicians.

**Disclosure of Interest:**

None Declared

